# Biphasic Double-Network Hydrogel With Compartmentalized Loading of Bioactive Glass for Osteochondral Defect Repair

**DOI:** 10.3389/fbioe.2020.00752

**Published:** 2020-07-02

**Authors:** Bingchuan Liu, Yanran Zhao, Tengjiao Zhu, Shan Gao, Kaifeng Ye, Fang Zhou, Dong Qiu, Xing Wang, Yun Tian, Xiaozhong Qu

**Affiliations:** ^1^Department of Orthopaedics, Peking University Third Hospital, Beijing, China; ^2^Engineering Research Center of Bone and Joint Precision Medicine, Ministry of Education, Peking University Third Hospital, Beijing, China; ^3^Center of Materials Science and Optoelectronics Engineering, College of Materials Science and Opto-Electronic Technology, University of Chinese Academy of Sciences, Beijing, China; ^4^Beijing National Laboratory for Molecular Science, State Key Laboratory of Polymer Physics and Chemistry, CAS Research/Education Center for Excellence in Molecular Sciences, Institute of Chemistry Chinese Academy of Sciences, Beijing, China; ^5^University of Chinese Academy of Sciences, Beijing, China

**Keywords:** osteochondral repair, biphasic scaffold, double-network hydrogel, dynamic cross linking, experimental research

## Abstract

Periarticular injury usually causes the defects of superficial cartilage and the underlying subchondral bone. Although some efficacious outcomes have been achieved by the existing therapeutic methods both in clinics and research, like symptomatic treatment, microfracture surgery, and tissue engineering technology, they still present specific disadvantages and complications. To improve this situation, we designed a biphasic (bi-) scaffold aiming to repair the structure of cartilage and subchondral bone synchronously. The scaffold consisted of a superior double-network (DN) hydrogel layer and a lower bioactive glass (BG) reinforced hydrogel layer, and the DN hydrogel included glycol chitosan (GC) and dibenzaldhyde functionalized poly(ethylene oxide) network, and sodium alginate (Alg) and calcium chloride (CaCl_2_) network. To investigate its effectiveness, we applied this biphasic scaffold to repair osteochondral full-thickness defects in rabbit models. We set up six observation groups in total, including Untreated group, Microfracture group, BG only group, DN gel group, bi-DN gel group, and bi-DN/TGF-β gel group. With a follow-up period of 24 weeks, we evaluated the treatment effects by gross observation, micro-CT scan and histological staining. Besides, we further fulfilled the quantitative analysis of the data from ICRS score, O’Driscoll score and micro-CT parameters. The results revealed that neat GC/Alg DN hydrogel scaffold was only conductive to promoting cartilage regeneration and neat BG scaffold merely showed the excellent ability to reconstruct subchondral bone. While the biphasic scaffold performed better in repairing osteochondral defect synchronously, exhibiting more well-integrated cartilage-like tissue with positive staining of toluidine blue and col II immunohistochemistry, and more dense trabecular bone connecting closely with the surrounding host bone. Therefore, this method possessed the clinical application potential in treating articular injury, osteochondral degeneration, osteochondral necrosis, and sclerosis.

## Introduction

Periarticular osteochondral structure usually includes three layers from the surface: articular cartilage, calcified cartilage, and the underlying subchondral bone, which can be further classified into cortical and trabecular bone. These well-organized anatomical structures closely integrate with each other and behave as a functional and structural unit supporting regular weight-bearing and joint motion (Bian et al., 2016; Goldring and Goldring, 2016). Normally, this unit could be damaged by several key reasons as follows: (1) vertical violence of peripheral joint; (2) severe degenerative osteoarthritis; and (3) osteonecrosis and sclerosis of peripheral joint caused by bone infarction. Whatever the reason is, one will suffer progressive joint swelling, pain and loss of motor function once encountering osteochondral defect. In order to ameliorate persistent symptoms and improve joint function, clinicians may choose some available conservative treatments such as non-steroidal anti-inflammatory drugs, pain killers, and surgical therapies including microfracture surgery, autologous chondrocyte implantation, and osteochondral auto/allograft transplantation, etc. Among the surgical options, microfracture surgery has been mostly applied in clinics. The prosthetic joint replacement will become the last-step for alleviation of severe symptoms. But the newly formed tissue regenerated via the existing repairing surgery is confirmed as a mixture of fibrous tissue and fibrocartilage mostly, and the subchondral bone cannot be sufficiently reconstructed (Mahmoud et al., 2017; McCarrel et al., 2017), which leads to the early good outcomes tending to deteriorate in long follow-up (Solheim et al., 2020).

Tissue engineering (TE) is considered as one of the most promising candidates to deal with the present dilemmas. Specific-designed substitute that provides sufficient functionalities comparable to the original tissue can be created by TE method. Thanks to constantly emerging advanced materials and techniques, TE-based therapies have achieved some effective outcomes in treating osteochondral defect (McCarrel et al., 2017; Rai et al., 2017; Nie et al., 2019). Nevertheless, numerous disadvantages still exist, such as tissue hyper-proliferation, dislocation of implants or cell death (Rai et al., 2017), besides, novel techniques are always hard to be directly translated into clinical application. In addition, articular osteochondral repair and functional restoration still face an essential problem nowadays, namely cartilage destruction always accompanies with subchondral bone injury, which can influence on the metabolism of the upper cartilage and further accelerate its deterioration. Though cartilage and subchondral bone are gradually being deemed as a whole construct when designing a therapeutic strategy, and many attempts have proposed that biphasic scaffold is a better choice to meet the different requirements for simultaneous healing of cartilage and bone (McCarrel et al., 2017; Rai et al., 2017; Filardo et al., 2018; Nie et al., 2019). But these approaches also possess some limitations. Some of the biphasic scaffolds were fabricated with different host materials, which resulted in weak bonding strength of both layers due to the absence of continuous phase (Rai et al., 2017), while some scaffolds depended on the cell insertion to play a more effective role (Kon et al., 2015).

In a previous research, we have successfully fabricated a novel biodegradable double-network (DN) hydrogel (Yan et al., 2017), consisting of glycol chitosan (GC) and dibenzaldhyde functionalized poly(ethylene oxide) (OHC-PEO-CHO) network, and sodium alginate (Alg) and calcium chloride (CaCl_2_) network (GC/Alg DN hydrogel). As reported, we evaluated its cytotoxicity, biodegradability, mechanical strength, and found its ability to promote chondrogenic differentiation and potential to repair cartilage defects. Compared with other hydrogels, the GC/Alg DN hydrogel could be prepared under physiological conditions, its mechanical strength could meet the requirements of supporting cartilage growth. When used as a scaffold to repair defective tissues, it would be completely degraded in the later stage of repair, and there would be no occupying phenomenon. And the degradation rate matched the growth rate of cartilage appropriately. In the current work, expecting to solve the weakness that the hydrogel itself does not have the ability to promote bone growth, we integrated bioactive glass (BG) particles inside the lower layer of GC/Alg DN hydrogel to form a biphasic scaffold and further added TGF-β1 to act as a positive reference. BG has exhibited its positive ability to enhance mechanical strength and promote bone regeneration in previous studies (El-Rashidy et al., 2017; Fu et al., 2018). By designing an animal model of full-thickness joint structure defect, including articular cartilage, subchondral cortical bone and the lower trabecular bone, we implanted the biphasic scaffold into the defect space in a surgically filled form to mimic a real clinical situation. We hypothesized that, in the early stage, surficial cartilage and the underlying bone could achieve homochronous regeneration due to the function of both GC/Alg hydrogel and BG particles. In the meanwhile, the scaffold could provide stable mechanical support for gradual cartilage crawling on its surface. In the later stage, with degradation of the DN hydrogel and slow-release of BG particles to accelerate bone regeneration, new cartilage can maintain structural and functional integrity with the help of underlying newly formed bone. In this way, the defect of both cartilage and subchondral bone can be repaired enduringly, with a potential of excellent long-term effects. We hope to use this experiment to explore the growth of joint structures and find an effective cell-free method to repair articular full-layered defects.

## Materials and Methods

### Materials

Poly(ethylene oxide) (PEO; MW = 2 kDa), glycol chitosan (GC, MW ∼ 250 kDa), and sodium alginate (MW ∼ 150 kDa, M/G ratio ∼ 1.6) were purchased from Sigma-Aldrich (St. Louis, United States). Benzaldehyde-capped poly(ethylene oxide) (CHO-PEO-CHO) and silica nanoparticles (average diameter of 12 nm) were synthesized according to our previous reports (Ding et al., 2010; Zhu et al., 2016, 2017). BG particles were synthesized according to our previous work (Ji et al., 2017). RPMI1640 medium, pancreatic enzymes, fetal bovine serum (FBS), penicillin-streptomycin solution, and phosphate-buffered saline (PBS) were purchased from Gibco (Grant Island, United States).

### Preparation of GC/Alg DN Hydrogel and the Biphasic Scaffold

**FIGURE 1:**
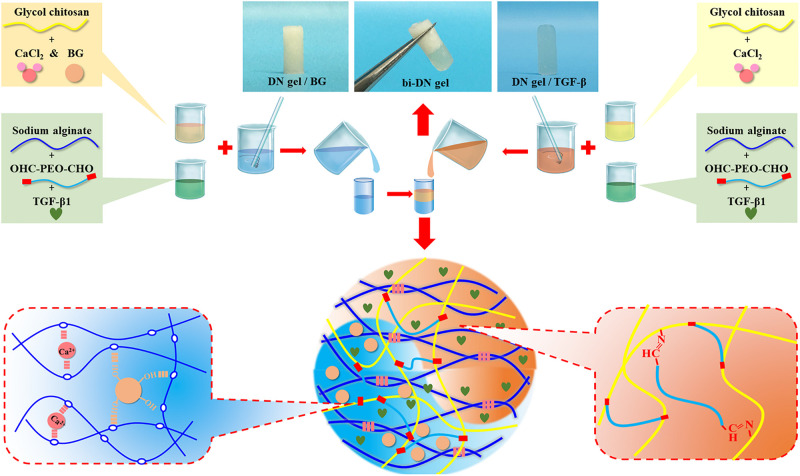
Illustration scheme of the formation of bi-DN gel.

An aqueous solution of GC and CaCl_2_ was prepared at a concentration of 4 wt% for each solute, while another solution of OHC-PEO-CHO and Alg was made with concentration of 6 wt% and 2 wt% respectively. Then partial GC/CaCl_2_ was mixed by desired amount of BG particles. In order to prepare a biphasic structured hydrogel scaffold, equal volume of the GC/CaCl_2_ and the OHC-PEO-CHO/Alg solutions were mixed together under vigorous vortex for 30 s and the mixture was quickly injected into a cylinder-shaped mold with a 4 mm inner-diameter, followed by a subsequent injection of a mixture of GC/CaCl_2_/BG and the OHC-PEO-CHO/Alg dispersions at ambient temperature. The mold was then removed a few minutes after the injection to allow the gelation of the scaffold. Upon a well-controlled injection volume, the obtained biphasic scaffold, named as bi-DN gel scaffold had an overall thickness of 4 mm, consisted of two 2 mm-thick hydrogel layers respectively containing BG particles and not containing BG. Such a topology was according to the previous studies on cartilage regeneration of osteochondral tissue on rabbit model (Kitamura et al., 2011; Higa et al., 2017). For the loading of growth factor (TGF-β1, 100 ng/mL), the bioactive molecules were just dissolved in either solution before the injection into the mold. The harvested scaffold samples were stored at 4°C under sterile condition for further use. The preparation process was illustrated in Figure 1.

### Characterizations

Surface morphology was observed using a Quanta-250 scanning electron microscope (SEM, Hitachi SU8000) Samples were prepared by freeze-fracture of wet samples through immersing the hydrogel pieces into liquid nitrogen, fractured and sputter coated with gold. XRD measurements were performed on a Rigaku D/MAX 2500 PC with Cu Kα radiation (λ = 1.54 Å), operated at 40 kV and 200 mA. The powder XRD patterns was collected at a scanning rate of 4°/min over a diffraction angles (2θ) from 10° to 70°. Infrared spectroscopy was recorded using a Bruker tensor 27 FT-IR spectrometer. Freeze-dried samples were pressed with KBr and scanned from 4000 to 400 cm^–1^. As described above, the mixed solution/dispersion was vortexed for a given time. Then the mixture was poured onto the bottom plate (diameter 25 mm) of a rheometer (Thermo Haake MARS). Time sweeping and frequency sweeping were carried out at 37°C with a gap of 1 mm at a shear strain of 1% amplitude which was predetermined in the linear viscoelastic region.

### *In vitro* Mineralization

Molded DN gel sample (1 mL) was immersed in 4 mL of simulated body fluid (SBF) at 37°C, and the SBF was changed every 2 days. At predesigned time points, the samples were washed gently with DDI water and lyophilized for further characterizations.

### Cytotoxicity

Cytotoxicity was assessed by CCK-8 assay on L929 cell line. DN gel samples (0.2 mL) were immersed into 0.8 mL of RPMI1640 culture medium at 37°C for 24 h, 48 h, and 72 h. The leach liquor was collected to incubate the cells. L929 cells were seeded in 96-well plates at a density of 1 × 104 cell/well. After 24 h incubation, the culture media was removed by the sample solutions, and the plates were incubated at 37°C for 24 h. 100 μL of freshly prepared medium with 10% CCK-8 reagent solution was added to each well and incubated for 1 h before measurement at the absorbance of 450 nm using a microplate reader (Thermo MULTISKAN MK3).

### Animal Grouping, Model, and Harvesting Samples

The study animals were bred at the Department of Laboratory Animal Science of Peking University Health Science Center. Animal selection and feeding, surgical protocol, and preparation procedures were carried out in accordance with the guidelines of Good Laboratory Practices (GLP) regulations and were approved by the Ethical Committee of Laboratory Animal Science Research (No. LA201708). A total of fifty 6-month-old male New Zealand white rabbits (weighting from 3.0 to 3.5 kg) were enrolled in this study. Both knees of each rabbit were included in the whole process, so we obtained one hundred experimental legs in all. They were divided into six groups, including Untreated group (defect was left empty without any intervening method, *n* = 15), Microfracture group (defect was repaired by microfracture technique, *n* = 15), BG only group (defect was repaired by BG scaffold implantation, *n* = 15), DN gel group (defect was repaired by GC/Alg DN hydrogel scaffold implantation, *n* = 15), bi-DN gel group (defect was repaired by the novel biphasic scaffold implantation, *n* = 15), and bi-DN/TGF-β gel group (defect repaired by bi-DN hydrogel plus TGF-β1 scaffold implantation, *n* = 15). And the remaining ten legs were regarded as additional supplements in case of probability for sample degeneracy.

All rabbits were preserved for 1-week acclimation period to confirm rabbits’ health and normal patellofemoral joint movement, and before surgery, all rabbits got food and water removed for 24 h to relieve gastrointestinal reaction during surgical procedure. General anesthesia was completed by the ketamine hydrochloride (50 mg/kg, IM) and fentanile (0.17 mg/kg, IM). After being postured appropriately, their knees were shaved carefully and cleaned with iodophor for three times, and sterilely draped. Routine surgical protocol was carried out by the lateral parapatellar approach. The patella was everted laterally, and intra-articular structures were thoroughly inspected for any abnormal conditions, such as infection and deformity. After confirmation of normal intra-articular environment, the knee joint was fully flexed and a surgical corneal trephine was applied to create the uniform cylindrical defect at the peak area of trochlear groove. Then the surgical field was continuously flushed with sterile saline solution to minimize heat generated by the drilling process. The full-thickness osteochondral defect model (3.0 mm in diameter, 4.0 mm in depth) was created, and the modeling process was precisely guided by the scale line on a corneal trephine. According to the preoperative plan, we implemented the specific intervention for each group. In Untreated group, the defects were kept empty along with washing carefully by saline solution. In Microfracture group, we successfully performed the microfracture technique by needle drilling until blood overflowed from the medullary cavity covered defects absolutely. In other experimental groups, the defects were repaired by implanting the corresponding scaffolds. After ensuring the stability of scaffold, joint capsule and overlying muscles and skin were carefully sutured. Rabbits were housed in separated cages and allowed to move without limitation. In the early 5 days after surgery, intramuscular antibiotics (cefazolin sodium, 0.2 g/kg) were administered to prevent potential infections. During follow-up, rabbits’ general conditions were collected with time, including range of knee motion, walking gait, dietary and mental status, and wound recovery. At 4, 12, and 24 weeks postoperatively, rabbits were sacrificed by means of euthanasia and their distal femurs were cut off to finish further steps. 5 samples were harvested at each time point in each group; timely replacement would be completed once unexpected failure or death happened.

### Gross Observation

After harvest, samples were observed for evidence of severe inflammation, extensive fibrosis, and scaffold ectopia. Coloration, luster, irregularity, any depression or bulging of regenerated tissue and inherent cartilage, and ectopic osteophyte were also carefully examined. Besides, we also applied the International Cartilage Repair Society (ICRS) score to quantitatively evaluate the macroscopic cartilage regeneration in aspects including degree of defect repair, integration to the border zone, macroscopic appearance and overall repair assessment.

### Micro-CT Analysis

Micro-CT scan was performed by the microtomography scanner (INVEON, Siemens, Germany) to visualize the mineralized tissue in growth inside the defects (5 samples in each group). To quantify the amount and quality of newly formed trabecula, we chose a region of interest (ROI) inside the defects with three-dimensional reconstruction system. The microstructure parameters, including bone volume fraction (BV/TV), trabecular number (Tb.N), trabecular thickness (Tb.Th), and average space distance between trabecular structures (Tb.Sp), were took notes and analyzed.

### Histological Evaluation

The eligible samples were fixed in 10% paraformaldehyde for 3 days and then decalcified in 10% EDTA solution. Following appropriate decalcification, samples were dehydrated, embedded in paraffin, cut into 5-μm slices sagittally, and stained with Hematoxylin and Eosin (H&E), and Toluidine Blue (TB). Besides, collagen type II (Col II), which is one of the most important components of hyaline cartilage, was assessed by immunohistochemical (IHC) staining. The H&E staining was good at recognizing the cellular density, morphology, type and distribution; in addition, it displayed the degradation process of the scaffold. The content and distribution of the glycosaminoglycans (GAGs) and Col II in the extracellular matrix (ECM) could be evaluated by TB and IHC staining. Based on the O’Driscoll histological grading scale (El-Rashidy et al., 2017), the regenerated tissues were graded blindly by three independent researchers for the overall evaluation of tissue morphology, matrix staining, surface regularity, structure integrity, thickness of neo-formed cartilage, bonding to adjacent cartilage, chondrocyte clustering, hypocellularity, degenerative changes in adjacent cartilage and inflammation in the defects. Total score ranges from 0 to 26, and higher points indicate better repair effect.

### Statistical Analysis

Statistical analysis was performed by SPSS 20.0 software. Difference in sum of ICRS score, O’Driscoll score and micro-CT parameters were compared by the Kruskal-Wallis Test. *P* < 0.05 was defined as statistically significant difference. The results were displayed in a graph.

## Results

### Preparation of Biphasic Hydrogel Scaffold (bi-DN gel)

**FIGURE 2:**
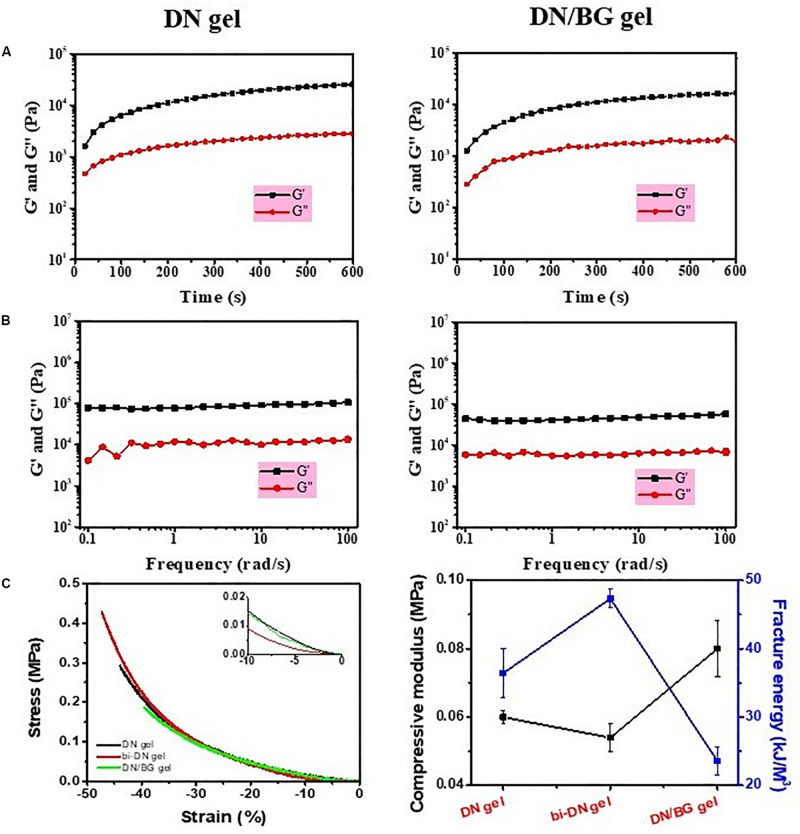
**(A)** Time sweeping and **(B)** frequency sweeping curves of DN gel and DN/BG gel; **(C)** Compressive properties of DN, bi-DN and DN/BG gels.

The synthesis of neat GC/Alg DN hydrogel has been studied previously (Yan et al., 2017). The crosslink of the hydrogel was achieved via the combination of benzoic-imine dynamic covalent bonding and ionic interaction, which endowed the injectability and the self-healing ability of the hydrogels. Besides, the double-network structure contributed to improving the mechanical properties of the gel, especially under compression, when compared to the present single-network gels. In this work, the capacity of the DN gel for the incorporation of BG microspheres was further investigated. The sol-gel transition of the composite gelling system was first monitored using a rheometer, by which it showed that with 3 wt% of BG particles, the gelation time of the sol solution containing 3 wt% of GC, 3 wt% of sodium alginate, 2 wt% of benzaldehyde capped PEO and 1 wt% of CaCl_2_ was slower than that without BG at 37°C, i.e., 140 s vs. 110 s (Figure 2A). Meanwhile, the shear modules, i.e., G’ and G”, of the BG loaded hydrogel, denoted as DN/BG gel, were also slightly lower than those of the pure DN gel (Figure 2B). The phenomenon could be attributed to the barrier effect of the inorganic particles on the close of the polysaccharide chains, and hence decreased the crosslink degree to the DN/BG gel. However, the injectability of the DN/BG gel was not influenced with the inorganic component, which allowed the molding of the composite hydrogel scaffolds. Besides, with the dynamic nature of the crosslinks, the composite hydrogel still possessed self-healing characteristic, which favored the construction of hierarchical structured bulk gels. It helped the fusion of hydrogel blocks with different functional materials or different crosslink degrees to build a multi-compartmental structure in three-dimension. As demonstrated in Figure 1, double-layered hydrogel cylinder was fabricated as the simplest example by a step-by-step way of injection of the DN/BG gel and the neat DN gel respectively into a cylinder-shaped mold. The biphasic hydrogel, denoted as bi-DN gel scaffold, showed a clear and firm interface. The loading of BG was expected to help the regeneration of bone tissue while the neat hydrogel, being a viscoelastic matter, would benefit the formation of cartilage tissues at articular surface (Yan et al., 2017).

The mechanical property of the bi-DN gel was then assessed as shown in Figure 2C. The compressive moduli of individual DN/BG gel was higher than that of the pure DN gel, due to the reinforcement function of the particles in the hydrogel matrix (El-Rashidy et al., 2017; Fu et al., 2018). However, it was found that the fracture energy of the composite hydrogel was much lower, attributed to the smaller break point (ca. 40%, Figure 2C), which was reasonable for that the inclusion of hard particles in a hydrogel matrix made the gel more brittle (Filardo et al., 2014). Also from Figure 2C, it was somehow interesting to see that the bi-DN gel afforded the largest deformation against compression, i.e., ca. 47%, and thus, gaining the highest fracture energy among the three samples, although it had the lowest compressive modulus. Considering that the bi-DN gel was composed of two different bulk gels, such results could be just explained by the asymmetric deformation on the DN and the DN/BG parts under a compressive stress. During the compression, it was observed that the transitional interface between the DN and the DN/BG parts kept stable, indicating the strong evidence on the affinity of the healed interface, which could be able to balance the energy dissipation between the two parts.

**FIGURE 3:**
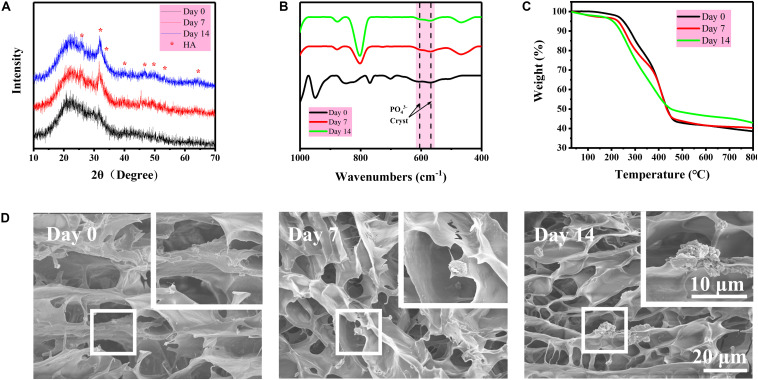
**(A)** XRD diagrams of freeze-dried DN/BG gel after different days of mineralization in SBF; **(B)** FTIR spectra of freeze-dried DN/BG gel after different days of mineralization; **(C)** TGA traces; and **(D)** SEM images of freeze-fractured DN/BG gel after different days of mineralization.

Hydrogel is a water-rich scaffold. Herein, the incorporation of BG particles is to endow the osteogenic activity for the repair of subchondral bone that may be damaged along with cartilage during articular injury. Therefore, the mineralization of BG inside the DN/BG gel was checked *in vitro* in simulated body fluid (SBF). The formation of hydroxyapatite (HA) was revealed from the XRD, FTIR spectra, and thermogravimetric analysis (TGA) (Figures 3A–C), in which classic diffraction peaks of HA and typical absorbance of phosphate groups became more obvious with the mineralizing time up to 14 days. SEM observation (Figure 3D) showed that particulate components could be distinguished in the porous structure of the freeze-dried DN/BG gel. And larger inorganic aggregates were then viewed in the sample at longer time point, e.g., 14 days. The observation was in agreement with the XRD and FTIR data, indicating the conversion of HA from the BG particles. Nevertheless, since the loading of BG was at low level, i.e., 3wt%, either the BG or the resultant HA remained as a dispersed phase in the hydrogel. It is certainly sure that the size of the inorganic particles, i.e., ca. 5 μm in this work, was far outweighing the mesh size of DN gel. Thus, there was no possibility for the inorganic component to migrate to the other part of the bi-DN gel across the interface. The compartmentalization can guarantee the realization of the functions in each individual layer.

**FIGURE 4:**
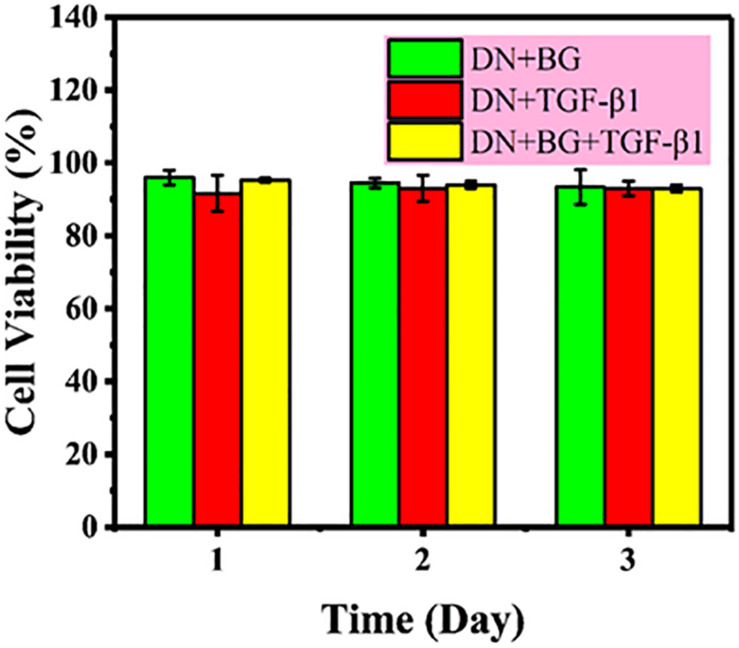
Cytotoxicity of leach liquor of DN gel with BG, DN gel with TGF-β1, and DN gel with BG and TGF-β1 on L929 cell line.

The safety of the bi-DN gel was first assessed using CCK-8 assay on L929 cell line with the leach liquor and results were showed in Figure 4. Percentage cell viability was expressed relative to the negative control (untreated cells) and the positive control (10% CCK-8 in medium), show non-toxicity as a function of extraction time up to 72 h.

### Osteochondral Defect Repair by bi-DN Gel Scaffold on Rabbit Model

Totally six groups of animals were under investigation for 24 weeks. Two rabbits were excluded from this study due to wound infection, and alternative samples were supplied immediately. Surgical incisions healed normally at about 14 days after surgery. All surviving rabbits expressed different levels of claudication at 1 month after surgery, and they gradually returned to normal with time.

#### Gross Observation

**FIGURE 5:**
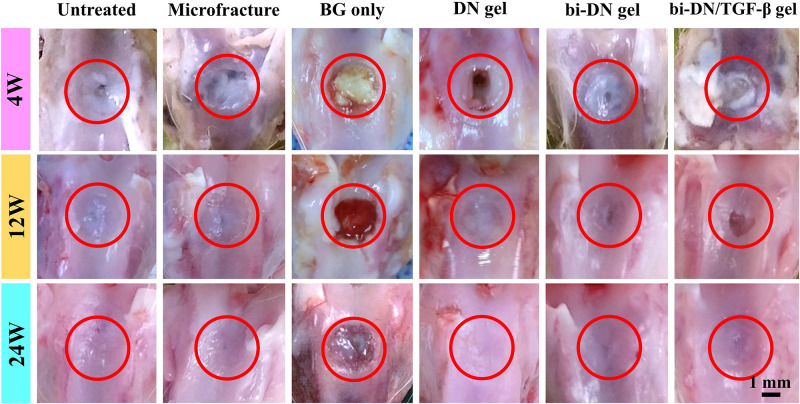
Gross observation of defect surface at 4, 12, and 24 weeks after surgery. The defect areas were highlighted by red circles (BG, bioactive glass; DN gel, GC/Alg double network hydrogel; bi-DN gel, double-layered DN gel).

At 4 weeks after surgery, the defects on the joints were similar in gross appearance for all groups (Figure 5). Only small part of neo-tissue appeared at marginal area, while the rest area kept empty, leaving the defects at the trochlea groove still obvious. At 12 weeks, it was a little bit surprising that fewer cartilage tissue was found in the group treated just using the BG particles (BG only group). Instead, randomly distributed fissures were observed in the surface and the margin. However, in the hydrogel treated groups, together with the microfracture group and the negative control group, the wounds were already partially covered by the regenerated tissue, leaving some un-filled area at the central of the defects. Particularly in the hydrogel treated groups (DN gel group), the regenerated tissue was relatively smooth, transparent and integrated well with the nature cartilage, regardless the inclusion of a DN/BG gel layer. Besides, the regenerated tissue was reddish-white, which seemed like the mixture of fibrous and fibrocartilaginous tissue. Nonetheless, the neo-tissue in the hydrogel treated groups was still thinner than surrounding cartilage, especially in the groups without growth factor, so that the boundary at the marginal region was more obvious. At 24 weeks, the gross appearance of all groups, except for the BG group, became smoother and well-integrated. Still, the BG treatment failed in the regeneration of cartilage although the defect was covered by a thin layer of fibrocartilaginous-like tissue. Among the hydrogel-treated groups, the administration of TGF-β1 with the BG particles eventually achieved positive result. The neo-tissue demonstrated pearly white color and translucent superficial surface, and resembled well with the surrounding normal cartilage. Meanwhile, the treatment using DN gel and bi-DN gel also resulted in finely regenerated cartilage tissue, fully covering the defects, integrating with by the adjacent cartilage, showing the mildest abrasion degree than the control and microfracture groups.

#### Micro-CT Evaluation

**FIGURE 6:**
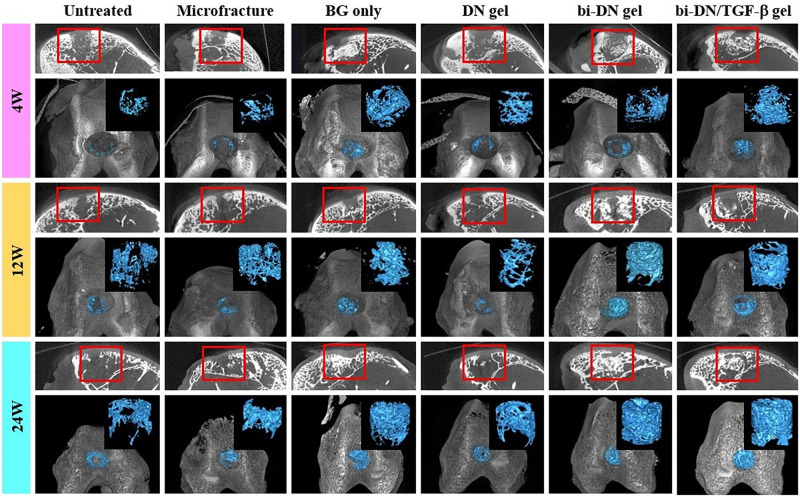
Three-dimensional reconstruction micro-CT images of sagittal plane, newly regenerated bone (blue tissues) and entire femoral condyle at 4, 12, and 24 weeks after surgery. The volume and structure of trabecula were displayed clearly. Red boxes can highlight the quality of new bone from the surface layer to the deep space (BG, bioactive glass; DN gel, GC/Alg double network hydrogel; bi-DN gel, double-layered DN gel).

Micro-CT images including three-dimensional reconstruction of sagittal plane, newly regenerated trabecular bone and entire femoral condyle, which were collectively dedicated to evaluate the quality of new bone formation (Figure 6). Overall, from 4 to 24 weeks, the new bone grew scatteredly from the margins to the middle in the Untreated, Microfracture and DN gel groups without uniform trabecular structures. While in other three groups, new bone could grow densely around the scaffold with the help of the promotion function from BG particles and interweaved with the surrounding normal trabecular bone. At 4 weeks after surgery, new bone hardly appeared in the defects of Untreated, Microfracture and DN gel group, while disturbed mineralization seemed the most obvious in other three groups. At 12 weeks, new regenerated bone could be confirmed at the marginal region in the surface layer of all defects, besides, the volume of new bone increased in the deep region compared with before. Part of new trabecular structures interconnected to each other clearly, but there existed some irregular new trabecular clustering desultorily. Interestingly, the three groups that were better at 4 weeks still exhibited excellent bone reconstruction effect at this time point. At 24 weeks, new regenerated bone almost filled the deep space of defects in BG only, bi-DN gel and bi-DN/TGF-β gel groups, thick trabecular structures closely interweaved together and mostly arranged in the same direction as normal bone. In addition, the new bone in the surface layer nearly connected as whole from both ends to middle. This could provide stable mechanical support for the new cartilage crawling on its surface. But in some marginal areas, the newly regenerated bone also existed with cluster and irregular structures. Whereas, in other three groups, the deep space of defects still kept empty mostly with low mineral density, and the new bone in the surface layer was just confined to the margin region.

(a) H&E staining;
(b) Toluidine blue staining;
(c) Col II immunohistochemical staining.

#### Histological Observation

**FIGURE 7:**
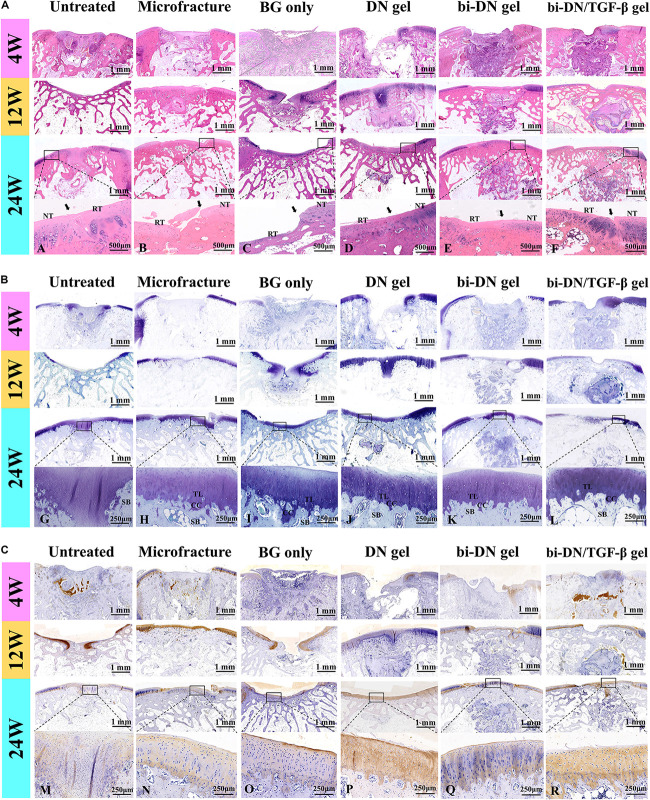
Histological evaluation of new cartilage and subchondral bone formation at 4, 12, and 24 weeks after surgery (NT, normal tissue; RT, repaired tissue; TL, tideline; CC, calcified cartilage; SB, subchondral bone; the arrows indicated the margins of the normal tissue and repaired tissue). H&E staining **(A)**, Toluidine Blue staining **(B)**, and Col II immunohistochemical staining **(C)** were integrally applied to recognize the cellular density, morphology, type and distribution, degradation of scaffolds, and to evaluate the structure, content and distribution of the glycosaminoglycans and Col II in the extracellular matrix (BG, bioactive glass; DN gel, GC/Alg double network hydrogel; bi-DN gel, double-layered DN gel). A–R indicated the enlarged observation of the corresponding region in the little box.

The tissue regeneration of the sections was monitored by H&E staining, TB staining and immunostaining of Col II (Figure 7). At 4 weeks, the histopathological sections inferred that the defects in all groups were mostly covered by immature fibrous tissue. Only a small amount of cartilage-like tissue was implied by the positive TB staining and the increased cell density at the edge of the defect in the bi-DN/TGF-β group. By the H&E staining, residual scaffolds could be clearly seen in the implanted areas of hydrogel treated groups. At 12 weeks, in the Untreated group, the cartilage loss was still replaced by fibrous-like tissue with low content of GAGs and Col II, whereas subchondral structure started to reconstruct, evidenced by the bone trabecula growth. Similar in the BG group, it showed the formation of subchondral bone together with the poor cartilage regeneration. On the other hand, the Microfracture group demonstrated some neo-cartilage tissue, with higher Col II and GAG expression, and meanwhile a smoother and more integrated surface of the defect was also observed. However, the growth of subchondral bone seemed slower than the BG group. Comparably, in the DN gel treated groups, the cartilage-like tissue had covered nearly the whole defect, supported by both the TB staining and the Col II distribution results. Similar in the bi-DN and bi-DN/TGF-β groups, although the cartilage layer was not yet completely repaired, the uncovered defects were much smaller. At this time point, the hydrogel residues could still be distinguished in all the gel treated groups. And notably, primary growth of subchondral bone was revealed in the bi-DN and the bi-DN/TGF-β groups. From the preceding gross observation (Figure 5), the defects of bi-DN/TGF-β gel group appeared more obviously than bi-DN gel group at 12 weeks after surgery, but according to the corresponding H&E staining in Figure 7A, the height of repaired tissue in bi-DN gel group was significantly lower than the surrounding normal cartilage, besides, poor Toluidine blue and Coll II immunohistochemical staining intensity indicated that the repaired tissue was mainly composed of fibrocartilage. While in bi-DN/TGF-β gel group, as showed by Figure 7A, the repaired tissue integrated more tightly with normal cartilage, and the height of repaired tissue was closer to the surrounding host cartilage. These staining characteristics collectively proved that the repaired tissue of bi-DN/TGF-β gel group was much closer to normal cartilage, and the implantation of TGF-β1 was helpful to promote cartilage regeneration to a certain degree. At 24 weeks, cartilage defect still existed in the Untreated group, while subchondral structure could be seen, but accompanied by considerable amount of cicatricial tissues, fibrous tissues and fat cells. In the BG group, the defect was covered by irregular fibrocartilage-like tissue with less positive expression of TB and Col II staining, but the regenerated subchondral bone seemed denser than in the Untreated group. In the Microfracture group, the GAG content, as represented by the TB staining, was more positive, in both the cartilage and the bone areas. However, it was obvious that the formed cartilage layer was discontinuous, showing a crack on the edge to the original tissue. The expression of Col II on the cartilage layer was also less pronounced, implying a fibrocartilage-like structure. Nonetheless, the subchondral defects were quite filled by plenty amounts of new bone, attributing to the favorable blood supply. The statuses of cartilage and bone regeneration were typical to the microfracture treatment, which was considered as a gold standard. At 24 weeks, as expected, cartilage-like tissue had completely filled the defects in the hydrogel treated groups, having good integrity with the surrounding nature tissue. The cartilaginous extracellular matrix could be identified by the TB staining and the level of Col II expression (Figures 7A,G–L,M–R). The clearer and mature structure of tideline and calcified cartilage was detected (Figures 7A,G–L). At the same time, the scaffolds had seriously degraded, especially in the DN gel groups, where a cavity could be seen under the newly generated subchondral bone. In contrast, although residual scaffold was still recognized in the bi-DN and the bi-DN/TGF-β groups, both the density as well as the thickness of the subchondral tissue was higher, which was comparable to the BG group, an indication of the bioactivity of BG. This proved that the double-layered scaffolds had favored the generation of both cartilage and bone tissue. Besides, it was also noticed that with the growth factor, more features of hyaline cartilage were revealed in the bi-DN/TGF-β group. In terms of the integration between the repaired tissue and surrounding normal cartilage, as showed by the enlarged margins in Figure 7A. Figures 7A–F, they integrated smoothly without obvious crack and collapse appearing in DN gel group. In bi-DN gel and bi-DN/TGF-β groups, the height of the repaired tissue had been close to the normal cartilage, but some cracks still existed. While in Microfracture and BG only groups, the repaired tissue collapsed and the height of new tissue was significantly lower than the surrounding normal cartilage.

#### Quantitative Analysis

**FIGURE 8:**
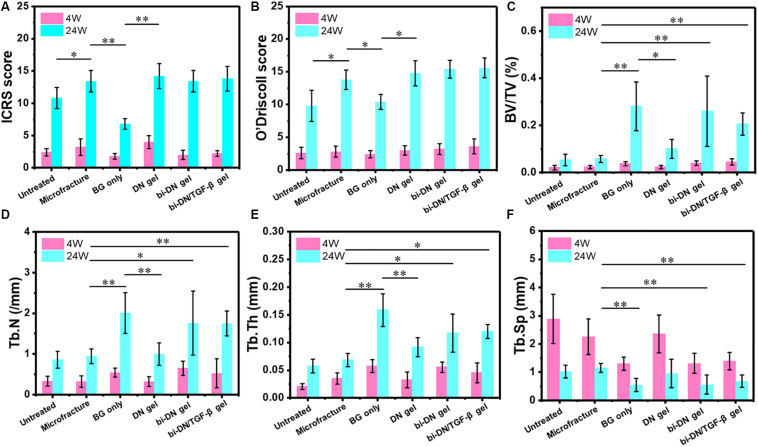
Quantitative analysis graphs of ICRS score **(A)**, O’Driscoll score **(B)**, BV/TV **(C)**, Tb.N **(D)**, Tb.Th **(E)**, and Tb.Sp **(F)** at 4 and 24 weeks after surgery. Significant differences were marked (BG, bioactive glass; DN gel, GC/Alg double network hydrogel; bi-DN gel, double-layered DN gel; *P < 0.05 and **P < 0.01).

The results of ICRS score, O’Driscoll score and micro-CT data were displayed in Figure 8. For statistical analysis, on one hand, we compared the differences in sum of ICRS score, O’Driscoll score and micro-CT parameters at 24 weeks after surgery between two adjacent groups in the histogram. On the other, we set the Microfracture group as the reference because its most wide application in clinics, and further compared the results of other groups with it. The statistical result could help to identify the differences between traditional method and this novel method for treating articular osteochondral defects. According to the results, as time went by (from 4 to 24 weeks), the parameters of ICRS score, O’Driscoll score, BV/TV, Tb.N, and Tb.Th displayed an increasing tendency, indicating that both cartilage and subchondral bone gradually regenerated in all groups. Besides, the decreasing tendency of average space distance between trabecular structures (Tb.Sp) reflected that newly formed trabecular bones interconnect and integrated with each other more closely. In terms of ICRS score, the BG only group was significantly lower than Microfracture and DN gel groups (*P* < 0.01), and no statistical difference was acquired among the Microfracture, DN gel, bi-DN gel, and bi-DN/TGF-β gel groups (*P* > 0.05). But the score of Microfracture group was higher than Untreated group with a statistical difference (*P* < 0.05). In the light of O’Driscoll score, the results were almost consistent with ICRS score. The score of Untreated and BG only groups was relative lower compared with Microfracture and DN gel groups (*P* < 0.05), which indicated that leaving the defect empty and implanting a neat BG scaffold were not beneficial to cartilage repair. Yet though the O’Driscoll score presented a gradual increasing trend in the order of Microfracture, DN gel, bi-DN gel, and bi-DN/TGF-β gel group, no significant statistical difference existed (*P* > 0.05). When it came to BV/TV, Tb.N, Tb.Th, and Tb.Sp, at 24 weeks, the three groups of Untreated, Microfracture, DN gel were relatively worse than other three groups (*P* < 0.05 or *P* < 0.01). The same statistical law indicated that new bone could regenerate better in the groups containing BG. But it was also interesting to note that, when comparing bi-DN/TGF-β gel and bi-DN gel groups, though the results exhibited some positive effects, the implantation of TGF-β1 did not increase the ICRS score, O’Driscoll score and micro-CT parameters to the extent of a statistical difference (*P* > 0.05).

## Discussion

Objectively, articular cartilage and subchondral bone are two dissimilar tissues as to their biochemical constitutions, mechanical properties, architectures and intrinsic healing capacities (Neufurth et al., 2014), and thus, simultaneous rehabilitation of osteochondral unit remains a prominent challenge in clinics. On one hand, the neo-cartilage regeneration cannot match the underlying mechanical support ideally, which can cause the gradual crack and collapse spreading on the neo-cartilage under the repeated joint abrasion and compression. On the other hand, failure to form stable integration between regenerated tissue and surrounding host tissue will lead to cartilage deterioration through inhibition and synthesis of the important components of cartilage matrix such as Col II and aggrecan during the long-term period (Goldring and Goldring, 2016). In some previous studies, many researchers attempted to focus on the reconstruction of cartilage layer alone, whereas accumulating evidences have shown the close correlation of surficial cartilage with underlying subchondral bone, which is involved in the pathogenetic process and may affect treatment results (Filardo et al., 2014). Due to the limited ability of cartilage to heal spontaneously, progressive degeneration and osteochondral defect may become inevitable once articular cartilage encountered the damage. Just like the progression of osteoarthritis, cartilage loss could occur in the same regions of the joint with the changes in subchondral bone (Goldring and Goldring, 2016). Currently, in order to overcome the limitations of the existing treatments as mentioned above, scaffold-based method is gradually emerging as a hopeful alternative and taking positive effects in repairing osteochondral defects (McCarrel et al., 2017; Rai et al., 2017; Saltzman and Riboh, 2018; Airapetov et al., 2019). In particular, multifunctional biphasic scaffold, which is composed of a cartilage phase and a subchondral bone phase, has attracted more attentions due to its appropriate and adjustable properties for each specific environment (Ruan et al., 2017; Zhang et al., 2017; Nie et al., 2019). But most studies are at exploratory stages, the optimal therapeutic option still needs more innovative and original attempts from researchers and physicians.

In this study, we applied an absorbable biphasic construct to repair osteochondral defect in a rabbit model. The double-layered structure was divided into an upper layer consisted of GC/Alg DN hydrogel intending to favor chondrogenesis and a lower layer composed of GC/Alg gel with BG particles to meet special requirement for subchondral bone growth. These two layers produced by the same host material, aiming to hold a tight interfacing conjunction between them. We have demonstrated the cytocompatibility and chondrocyte-induction of the GC/Alg DN gel in our previous study (Yan et al., 2017). But the ability of this DN gel for treating osteochondral tissue was also demonstrated, that is, a hydrogel may not have any osteogenic activity (Khorshidi and Karkhaneh, 2018). As seen in the presented data, thinner subchondral bone was resident in the DN group (Figure 6). In clinical practice, this may influence the stability and durability of repaired cartilage layer for patients, and further result in a mild collapse and rough surface of the joint.

So far, various strategies have been attempted for improving osteogenic capacity of hydrogel scaffolds, including the addition of growth factor and the corporation of functional materials like BG and hydroxyapatite. Since hydrogel is an ideal carrier of bioactive molecules, loading of inorganic components is considered as an efficient way to help the mineralization for the bone generation. Nevertheless, herein we observed that the osteogenic process, if mediated by BG particles alone, could disturb the formation of cartilage tissue (Figures 5, 7). Given the layered structure of articular surface, it should be preferable that the scaffold has a hierarchical architecture that enables to adapt the specific requirements for either bone or cartilage growth. The bi-DN gel scaffold was thus designed, based on the dynamic crosslink mechanism, which benefited the formation of a continuous matrix, where the BG particles could be restricted by the polysaccharide mesh in the bottom layer. Meanwhile, the loading of growth factor to the scaffold was not affected.

Using the bi-DN gel scaffold, we proved that the defects were repaired mostly at 24 weeks, with better quality in comparison with both GC/Alg DN gel and the use of neat BG scaffold. Specifically with the loading of TGF-β1, stronger positive TB and Col II were observed, suggesting the characteristics of cartilage-like tissue formulation (Reyes et al., 2014; Zhou et al., 2017). By adjusting the degradation rate, to match the cartilage regeneration process, the hydrogel worked as a reservoir to control the release of the encapsulated bioactive factor and gradually took effects during the postoperative period. By using the biphasic scaffold, the mature tideline and subchondral bone were superiorly visible in both the bi-DN and bi-DN/TGF-β groups 24 weeks after surgery (Figure 7A,G-L). Tideline is a histologically distinct basophilic boundary between non-calcified and calcified cartilage, which is a marker for cartilage maturation and regeneration (Chen et al., 2011; Wu et al., 2014). Through the structure of tideline and calcification, the new cartilage and subchondral bone was connected tightly. In addition, high-density subchondral bone layer was also harvested in these two groups compared to the DN gel treated group. According to the micro-CT calculation, the difference became statistically significant after BG attrition in the aspects of BV/TV, Tb.N Tb.Th, and TB.Sp. Whereas the newly formed fibrocartilage tissue after microfracture surgery, an universally applied method in clinical settings, occurred obvious collapse with the breakage of marginal connection (Figure 7A). Without stable support from the underlying scaffold or bone, the incipient effects were difficult to sustain for a long time.

As shown by Figure 8, it was worthy to point out that the addition of TGF-β1 did not significantly promote the regeneration of cartilage and bone, without statistical difference between bi-DN gel group (*P* > 0.05). The reason might be related its dual-directional function in regulating the growth and regeneration of cartilage and bone. TGF-β1 could play a positive role via promoting the proliferation and differentiation of mesenchymal stem cells (MSCs) as well as the synthesis and secretion of the specific cell matrix (Zhen et al., 2013; Reyes et al., 2014; Zhou et al., 2017; Utsunomiya et al., 2020), but it could also exert an inhibiting effect. Some reports revealed that the concentration of TGF-β1 might play an imperative role, and local excessive concentration had the potential to inhibit the formation of new bone and cartilage, even resulting in osteoarthritis (Zhen et al., 2013; Utsunomiya et al., 2020). Further studies are required to profoundly understand this controversial issue that how to maximize the performance of TGF-β1 for osteochondral defect repair. Besides, the delayed release of BG particles embedded in the GC/Alg DN hydrogel might be other reason to cause lesser volume of newly formed trabecular bone compared with BG only group. Yet this was exactly the key guarantee for long-term effects of cartilage repair, and slow new bone promotion of BG particles could provide durable mechanical support for cartilage crawling, and further avoiding late cartilage collapse.

Through our study, the results definitely proved that cartilaginous and osseous layers should be regarded as a structural and functional unit to be repaired synchronously. During the progression of disease, these two compartments are intimately located and pathological changes take place in parallel. As shown in Figures 5, 7, 8, the scaffold-treated groups with adding BG particles into the underlying layer performed better in articular cartilage repair and long-term sustainability compared with the DN gel group. From the mechanical viewpoint, the cartilage transmits and distributes loads to the subchondral bone through the soft-to-hard interface during the chronically exposed to high mechanical stress. The underlying subchondral bone is responsible for maintaining the outline shape of articular bone and creating an appropriate biomechanical environment for the differentiation and development of new cartilage (Goldring and Goldring, 2016; Ansari et al., 2019; Fell et al., 2019). With respect to the biochemistry crosstalk, some small molecules can transit between two layers, and meanwhile the nutrient substance and metabolic waste can be exchanged by interlinked vessels and pores. Imhof et al. once claimed that more than 50% of the glucose, oxygen, and water requirements of cartilage are provided by perfusion from the subchondral vessels (Imhof et al., 2000). At the cellular level, there is mounting evidences that normalization of chondrocytes and osteoblasts behavior may influence each other (Valverde-Franco et al., 2012; Findlay and Atkins, 2014). It was highlighted that, in a coculture study, the excision of subchondral bone from articular cartilage resulted in an increase in chondrocyte death at 7 days (Amin et al., 2009).

## Conclusion

In summary, we demonstrated that a double-layer structured, double-network hydrogel-based scaffold, designed with chondrogenic and osteogenic functionalities respectively, could be implanted by a one-step surgery, and hence accelerated the growth of cartilage and bone tissue simultaneously in the anatomical space of damaged articular defect on a rabbit model. It was believed that this method owned the clinical application potential in treating articular injury, osteochondral degeneration, osteochondral necrosis and sclerosis. The construction of the bi-DN gel was facile and flexible, due to the utility of dynamic bonding and interaction as the crosslinks, harvesting the self-healing ability and easy-incorporation of inorganic or biomacromolecular components. The DN structure improved the mechanical property and prolonged the degradation of the scaffold, which benefited the regeneration of osseous tissues, by providing better supporting during the time-consuming period of trauma restoration. With these features, future work on the construction of 3D compartmental scaffolds using the DN gel would provide new opportunities for more efficiently treatment of periarticular defects.

## Data Availability Statement

The original contributions presented in the study are included in the article/supplementary materials, further inquiries can be directed to the corresponding authors.

## Ethics Statement

The animal study was reviewed and approved by the Ethical Committee of Laboratory Animal Science Research (No. LA201708).

## Author Contributions

BL and YZ finished this article. BL, YZ, TZ, SG, and KY finished the experiments together. FZ, DQ, XW, YT, and XQ designed this study. All authors analyzed the results together.

## Conflict of Interest

The authors declare that the research was conducted in the absence of any commercial or financial relationships that could be construed as a potential conflict of interest.
